# Comments on identifying causal relationships in nonlinear dynamical systems via empirical mode decomposition

**DOI:** 10.1038/s41467-022-30359-8

**Published:** 2022-05-23

**Authors:** Chun-Wei Chang, Stephan B. Munch, Chih-hao Hsieh

**Affiliations:** 1 National Center for Theoretical Sciences, Taipei, 10617 Taiwan; 2 Research Center for Environmental Changes, Academia Sinica, Taipei, 11529 Taiwan; 3 Ecology and Evolutionary Biology, University of California, Santa Cruz, CA 95060 USA; 4 Institute of Oceanography, National Taiwan University, Taipei, 10617 Taiwan; 5 Institute of Ecology and Evolutionary Biology, Department of Life Sciences, National Taiwan University, Taipei, 10617 Taiwan

**Keywords:** Ecology, Statistics

**arising from** Albert C. Yang et al. *Nature Communications* 10.1038/s41467-018-05845-7 (2018)

The empirical mode decomposition (EMD) method proposed in Yang et al.^1^ fails to correctly identify causal relationships for a system of two independent variables driven by a shared external forcing (aka Moran effect). Using a simple, two-species Moran effect model (Fig. 1a), it is obvious that the EMD method erroneously concludes that *N*_*1*_ and *N*_*2*_ have a causal relationship (IMF 1 in Fig. 1b), although in fact they do not. This is because effects of external forcing were recorded in both *N*_1_ and *N*_2_ time series, and at least one IMF decomposed from *N*_1_ and *N*_2_ time series is associated with that external forcing. Therefore, removal of such IMF from *N*_1_ makes the remaining less coherent with *N*_2_, and vice versa. As such, EMD methods based on diminished coherence due to IMF removals fail to falsify spurious causations caused by sharing external forcing. In contrast, convergent cross mapping (CCM) correctly identifies the lack-of causal relationship between *N*_*1*_ and *N*_*2*_ (Fig. 1c). The efficacy of CCM to distinguish Moran effects depends on the strength of the shared external forcing. In extreme cases, when external forcing is too strong, *N*_*1*_ and *N*_*2*_ are synchronized (the pathological case, as noted in a previous study^2^). Methods to cope with such situation have also been developed^3,4^.

**Fig. 1:**
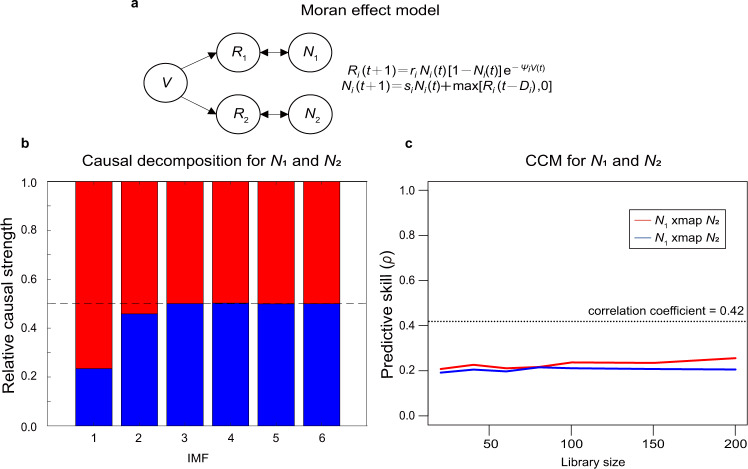
Causal decomposition fails to falsify spurious causations presented in Moran effect model. **a** Moran effect model is a 5-variate difference equation model in which variables *N*_1_ and *N*_2_ have no causal relationship, but have a significant correlation in their time series due to shared external forcing, *V*. We ran this model for 10,000 time steps with the parameter set [*r*_1_ = 3.4, *r*_2_ = 2.9, *ψ*_1_ = 0.5, *ψ*_2_ = 0.6, *s*_1_ = 0.4, *s*_2_ = 0.35, *D*_1_ = 3, *D*_2_ = 3, *R*_1_(0) = *R*_2_(0) = 1, *N*_1_(0) = *N*_2_(0) = 0.5], but retaining only the last 200 steps for analysis. Because of the strong correlation between *N*_1_ and *N*_2_. **b** The causal decomposition method, incorrectly concluded causation according to IMF 1 and 2, even though *N*_1_ and *N*_2_ do not interact. Here, causal decomposition is performed under 1000 ensemble EMD with noise level *r* = 0.085 selected based on the criteria of maximizing the separability but maintaining orthogonality of the IMFs, following the Matlab codes provided in Yang et al.^1^. In contrast, **c** CCM had no convergence (i.e., no improvement in CCM skill with increasing library size) in cross-mapping between *N*_1_ and *N*_2_, and thus correctly concluded no causation between *N*_1_ and *N*_2_.

Second, Yang et al.^1^ argued that CCM provides incorrect causal relationships. However, Yang et al.^1^ used CCM in a manner that the original authors^2^ did not intend, producing incorrect conclusions. Specifically, following McCracken et al.^5^, Yang et al.^1^ used correlation difference (X cross-map Y – Y cross-map X) as the definition of CCM causation (Fig. 3 in ref. ^1^) without examining the convergence of the cross-mapping skill; this is an incorrect definition. The correct definition of causation under CCM is improvement of cross-mapping skill with increasing time series length (i.e., convergence). In addition, as in real systems, CCM causation can be bidirectional^2^. Yang et al.^1^ used an incorrect definition (i.e., correlation difference) and incorrectly concluded that CCM misidentified the lynx versus hare and *Didinium* versus *Paramecium* interactions as top-down control systems and the Lotka Volterra predator–prey model and wolf versus moose interactions as either no or confusing causation. However, in each of these examples, CCM exhibits clear convergence with increasing library size in both directions. By using the convergence definition of causation in CCM, we concluded that these prey–predator systems exhibited bidirectional causation. That is, CCM correctly identified the reciprocal nature of predator–prey interactions in all of these systems (Fig. 5 in ref. ^1^). As such, we suggest any description of coupling in predator–prey systems as “directional” claimed by Yang et al.^1^ may be misleading (Fig. 3 in ref. ^1^) because predators causally influence prey by consuming them and prey causally influence predators by providing them the energy needed for population growth. Moreover, the relative strength of each direction can be quantified based on the rate of convergence^2,6^, with proper consideration of potential lagged effects^3^. We also disagree with the claim that CCM incorrectly identified causal coupling in white noise. Again, this also stems from using an incorrect definition of CCM. In contrast, we find no evidence of convergence when applying CCM to paired white noise signals (Fig. 2a) and a false positive rate consistent with *p* = 0.05 as the level of significance (Fig. 2b, c).

**Fig. 2:**
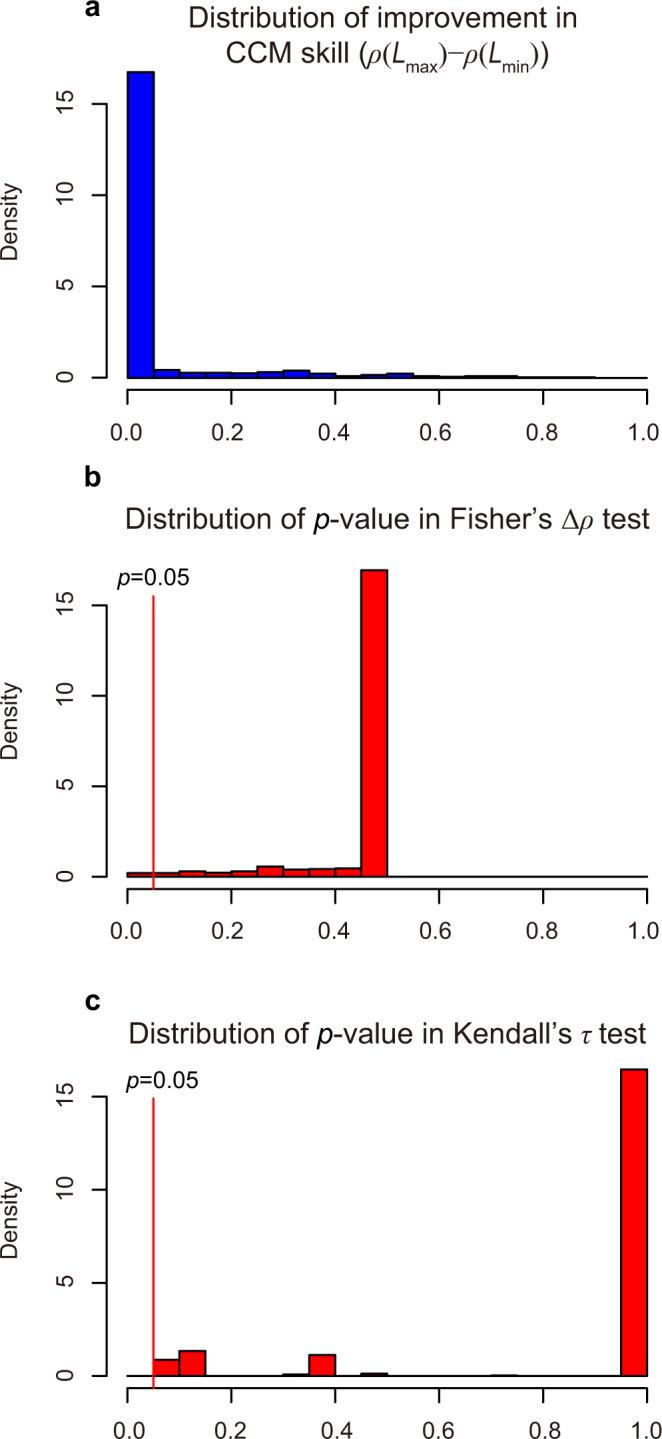
CCM analysis for paired white noises. White noise time series were generated from 10,000 simulations and all time series were trimmed to length = 10, following Yang et al.^1^. In total, we performed CCM analyses between 1000 random pairs of white noises. To evaluate convergence of CCM, we calculated three indices: **a** improvement in CCM skill from minimal (*L* = 2) to maximal library length (*L* = 10); **b** *p* value for testing the significance of the improvement in CCM skill using Fisher’s Δ*ρ* Z test; and **c** *p* value for testing the significance of monotonic increasing trend in CCM skill using Kendall’s *τ* test. In a majority of cases, improvements in CCM skill were very small and close to zero, indicating no convergence (**a**). As such, false positives in both Fisher’s Z test (**b**) and Kendall’s *τ* test (*p* < 0.05) (**c**) occurred, with very low probability. In summary, the probability of detecting spurious causation in paired short white noise was very low; this was opposite to conclusions of Yang et al.^1^ based on the incorrect definition of CCM.

Several additional misunderstandings about CCM in Yang et al.^1^ warrant clarification. (1) CCM does not rely on predictability as the criterion. Rather, CCM relies on information recovering^2,6^ that identifies whether the present state of an effect variable contains information about the present state of causal variables^7^ (i.e., nowcast) and thus enables CCM to identify simultaneous influences. (2) Oscillatory dynamics may confound the efficacy of CCM; however, methods to remove cycles or construct a null model that accounts for cycles (e.g., seasonality) have been developed^4,8–10^. (3) Yang et al.^1^ stated that “CCM is developed under the constraints of perfect deterministic system”; this is incorrect. In reality, real-world systems usually contain a deterministic skeleton convolved with stochastic processes^11^. In fact, modeling and empirical examples have demonstrated that CCM correctly identifies causation, even when stochastic processes are convolved with deterministic signals^2,6,12^.

It is noteworthy that interpretation of findings based on IMF subtraction needs to be done with caution. Yang et al.^1^ included a strong statement regarding interpreting their findings, stating that removals of causal-related IMFs enable us to exclusively recover intrinsic dynamics of the target series from the residual IMFs. Although this statement is correct when the system dynamic is a result of superposition of signals, it is not always correct for general dynamical systems in which causal influences cannot be easily separated from intrinsic dynamics. Using a simple example of prey–predator model, $\frac{dx}{dt}=\alpha x-\beta xy;\frac{dy}{dt}=\delta xy-\gamma y$, we have a clear expectation that the prey will grow exponentially in the absence of the predator, or at the very least, prey cannot oscillate. However, these expectations are not realized after subtracting causal IMFs; rather, the remaining components continue to cycle—quite at odds with intuition based on the statement provided by Yang et al.^1^. The salient point is that interpretation of the residual IMF is not as unambiguous as the original text implies. Certainly, IMF subtraction is not equal to mathematical subtraction. However, real-world biologists applying Yang et al.’s approach^1^ to predator–prey systems may be confused when interpreting the results according to Yang et al.’s statement about separability in causal inference. In fact, after subtracting the effect of the predator on the prey (e.g., Fig. 1c in ref. ^1^), prey continue to oscillate. Based on countless chemostat experiments, prey grown in isolation reach a steady state set by the rate of nutrient input and media outflow. Thus, sustained oscillations (e.g., remaining series after accounting for predator effect) suggest the existence of other factors. Assured by their IMF analysis that these oscillations are neither driven by the predator nor by its intrinsic dynamics, biologists might conclude that there must be another variable causing the oscillation (perhaps, time-varying fluctuations in resource availability or temperature) and fruitlessly search without success. Of course, they never find one, because the oscillation is artificially introduced by performing an additive decomposition on a non-separable system. Thus, we caution potential over-interpretation of the meanings of various IMFs in EMD.

To summarize, the EMD method of Yang et al.^1^ clearly works for systems in which superposition is obtained, but does not provide unambiguous results for non-separable, nonlinear dynamical systems.

## Reporting summary

Further information on research design is available in the Nature Research Reporting Summary linked to this article.

## Supplementary information

Reporting Summary

## Data Availability

Empirical datasets analyzed in this study are open-access and available by following the same instruction addressed in Yang et al.^1^. R source codes used to generate synthetic datasets from the Moran-effect model and white noises are available on GitHub, https://github.com/biozoo/CommentEMD.

## Code availability

Matlab and R source codes for conducting EMD causal decomposition and CCM analyses, respectively, are provided on GitHub, https://github.com/biozoo/CommentEMD.
